# Comparative oligo-FISH validates genome assemblies and delivers a precise karyotype for *Lens* Mill. species

**DOI:** 10.1371/journal.pone.0354141

**Published:** 2026-09-21

**Authors:** Alex Junior Aparecido Silvestrini, Larissa Ramsay, Eric Bishop von Wettberg, Kirstin E. Bett

**Affiliations:** 1 Department of Plant Sciences, College of Agriculture and Bioresources, University of Saskatchewan, Saskatoon, Canada; 2 Department of Agriculture and Life Sciences and Gund Institute for the Environment, University of Vermont, Burlington, United States of America; Hirosaki University Graduate School of Medicine, JAPAN

## Abstract

Chromosome structural rearrangements play a significant role in karyotype evolution and speciation. These rearrangements pose challenges for precise karyotyping and the assembly of a genus pan-genome for crops and their wild relatives. *Lens culinaris*, an important cool-season legume primarily grown in India and Canada, is a cultivated species with six wild relatives. All seven *Lens* species exhibit distinct chromosomal arrangements, which affect introgression and complicate the development of an accurate karyotype for the genus. Using gene synteny analysis between the cultivated *Lens* species and six wild relatives, we developed cross-species oligo-FISH (Fluorescent *in situ* hybridization) probes to *in situ* confirm genome assemblies and synteny. Chromosome spreads were used for oligo-FISH experiments, in which the DNA was denatured and a set of red and green oligo probes was hybridized to the metaphase chromosomes. The combination of both oligo sets/probes resulted in a distinct pattern for each *Lens* spp. chromosome, allowing the inference of a more robust karyotype for six *Lens* species. The karyotyping of *Lens* spp. confirmed the proper assignment of chromosomes in the genome assemblies and validated the rearrangements detected in the synteny analysis. The results attest to a higher sequence-level similarity among the closest related species despite the occurrence of several chromosomal structural changes among them. Oligo-FISH probes can be used in conjunction with plant genome assembly projects, supporting the delivery of a precise representation of the physical chromosomes of a species.

## Introduction

The genus *Lens* Mill. belongs to the family Fabaceae and legume tribe Vicieae and is comprised of seven taxa – the cultivated *Lens culinaris* and six wild relatives [1–3]. All *Lens* species are diploid, have 2n = 14 chromosomes [4] and are classified into four gene pools depending on the feasibility of crossing with the cultivated species. Lentil is an important pulse crop behind only chickpea and pea in cool-season legume global production [5]. The use of wild relatives for lentil breeding is a strategy for positive allele transfer [6–12] despite the issues that arise when trying to cross species from different gene pools. Chromosomal rearrangements are known to exist among *Lens* spp. [9,13,14] and pose a challenge for introgressions of desired alleles from donor to recipient due to the possibility of undesired allele combinations due to linkage drag [6,15]. *Lens* species are also of interest for chromosome evolution studies due to the ploidy level and chromosome number stability, coupled with the occurrence of several chromosomal changes.

Chromosome rearrangements have been reported in *Lens* species using chromosome pairing analysis of F1 interspecific at meiosis [16–18], genetic linkage maps [6,19,20] and more recently, synteny analysis [15]. *Lens* species karyotypes were previously built based on chromosome measurements and fluorescent *in situ* hybridization (FISH) using repetitive DNA sequences as marks [4,21,22]. Although very informative, none of those works confirmed the occurrence of the numerous rearrangements previously proposed, such as the formation of five bivalents on a hybrid between *L. culinaris* and *Lens orientalis,* suggesting at least one interchange among them [16] or an ancient translocation between *L. culinaris* and *Lens ervoides* that separates the two species suggested by genetic mapping [19]. There is still no consensus regarding the karyotype of the seven *Lens* spp., with authors proposing different karyotype formulas and different numbers and locations of ribosomal DNA sequences [23].

Karyotype studies can be a powerful tool to characterize chromosome rearrangements *in situ*, providing physical evidence of the chromosomal changes and overcoming synteny analysis limitations [24]. The recently developed oligo-FISH technique, using probes targeting unique sequences, can be a powerful resource for physically validating both the assignment of scaffolds to chromosomes and synteny analysis in genome assembly projects. The technique is based on the development of conserved oligo probes from genome assembly data covering specific genomic regions. The probes can be used in cross-species FISH experiments, allowing the inference of chromosome evolution, chromosomal compatibility, and building reliable karyotypes for the species analyzed [25–27].

We developed an oligo-FISH barcode system based on genome synteny analysis among the cultivated (*L. culinaris*) and wild *Lens* spp. We strategically defined genomic regions in the cultivated species genome assembly Lcu.2RBY [15] that would cover the maximum number of rearrangements between the cultivated and wild relatives. Our system confirms the rearrangements detected through the synteny analysis, as well as allows the validation of the genome assemblies at the chromosomal level and the quality assessment of both the genome assembly and the synteny analysis. The results are a reliable karyotype for six *Lens* species, which takes into account the rearrangements that shaped chromosome evolution across the genus and are very useful to assess chromosomal compatibility in plant breeding programs in the absence of genotype-specific genome assemblies.

## Materials and methods

### Plant materials and genome synteny analysis

*L. culinaris* cultivars CDC Greenstar and CDC Redberry were obtained from the Crop Development Centre (CDC) at the University of Saskatchewan. Accessions of *L. orientalis*, *Lens tomentosus*, *Lens odemensis*, *Lens lamottei* and *L. ervoides* were obtained from the Genebank at the International Center for Agriculture Research in the Dry Areas (ICARDA) and maintained in the CDC collection. *L. orientalis* BGE 016680 was the parent of a RIL population [28] obtained by the CDC from the Universidad de Leon, Spain. ILWL 25 is a *Lens nigricans* accession sourced from the Australian Grains Genebank (AGG) (S1 Table in S2 File).

The *L. culinaris* genome assembly Lcu.2RBY [15] is openly available at https://knowpulse.usask.ca/genome-assembly/Lcu.2RBY. The methodology used for assembling *L. ervoides* (Ler.1DRT) [15] *L. orientalis* (Lor.1WPS), *L. tomentosus* (Lto.1BIG), *L. odemensis* (Lod.1TUR), *L. lamottei* (Lla.1ESP) and *L. nigricans* (Lni.1VIC) is available at https://knowpulse.usask.ca/research-study/wild-Lens-genome-assemblies (Table 1). Synteny analysis between Lcu.2RBY/Lcu.1GRN and Ler.1DRT, Lor.1WPS, Lto.1BIG, Lod.1TUR, Lla.1ESP was done with MCScanX [29]. Genes with repeat-related functional annotation were removed from consideration, and the output was filtered by score for each genome pair using a custom Python script (Available at https://github.com/mcbbaker/genome-synteny). Results were visualized in Synvisio [30] (https://synvisio.github.io).

**Table 1:** *Lens* spp. accessions and respective genome assemblies used for oligo-FISH experiments. Probe prediction method from the reference genome, in which the probes were designed, and the BLASTN method in the wild *Lens* genomes.

Species	Accession	Genome assembly	Probe prediction
*Lens culinaris*	CDC Redberry	Lcu.2RBY	Reference
*Lens ervoides*	IG 72815	Ler.1DRT	BLASTN
*Lens culinaris*	CDC Greenstar	Lcu.1GRN	
*Lens orientalis*	BGE 016880	Lor.1WPS	
*Lens orientalis*	IG 72643	–	
*Lens odemensis*	IG 72623	Lod.1TUR	
*Lens tomentosus*	IG 72805	Lto.1BIG	
*Lens lamottei*	IG 110813	Lla.1ESP	
*Lens nigricans*	ILWL 125−0001	Lni.1VIC	

### Oligo probe development and analysis

Genomic coordinates spanning 16 chromosomal regions of the reference genome *L. culinaris* CDC Redberry (Lcu.2RBY) were chosen based on the gene-synteny predicted rearrangements using bedtools intersect [31]. Preferred Lcu.2RBY genomic coordinates, repeat annotation and the full assembly were shared with Arbor Biosciences (Ann Arbor, MI, USA) for probe development. The repetitive sequences in the Lcu.2RBY genome were filtered out, and the remaining sequences were divided into oligos of approximately 45 nt with a step size of 5 nt. These oligos were processed to remove duplicated oligos and oligos located within the likely centromeric regions to avoid centromere-specific sequences and possible unfiltered repeats. Two final oligo pools were synthesized – Lcu.2RBY_Gpool labelled with Alexa488 for green signal, and Lcu.2RBY_Rpool labelled with ROX for red signal.

The density of oligos in each probe for all wild *Lens* species was determined through BLASTN analysis. Both oligo pools (see Supplementary Tables) were used as a query against the assembled genomes of each wild *Lens* species using blastn -query <oligo pool sequences > -db <genomes in fasta format > -num_threads 60 -outfmt 6 -out <output file > . Oligo probe annotation was conducted by comparing gene-annotated genomic positions in Lcu.2RBY with predicted oligo probe positions using bedtools intersect [31]. All figures were generated using R package ggplot2 [32], Synvisio [30], JBrowse 2 [33] or IGV [34].

### Preparation of chromosome spreads

Seeds for each of the lines (S1 Table in S2 File) were germinated in the dark overnight, kept growing for 3 days and then treated with 1.18 mM Hydroxyurea (HU) solution for 18 hours to synchronize the cell cycle. Seedlings were left in Hoagland’s media without HU for cell cycle recovery, with times varying depending on species (S1 Table in S2 File), and subsequently treated with 15 µM Oryzalin for 4 hours to induce metaphase arrest. Roots were then fixed in Carnoy solution (ethanol:acetic acid 3:1, v/v) and stored at 10 ºC until further use. Root tips were subjected to cell wall digestion using a stock solution of enzyme mixture containing 100U/mL Pectinase Sigma-Aldrich, 200U/mL Cellulase Sigma-Aldrich and 3U/mL of Pectolyase Sigma-Aldrich at 37 ºC, with exposure times described in Supplementary Table 1, depending on the species. After enzymatic digestion, slides were prepared following the protocol described by Aliyeva-Schnorr et al., 2015 [35]. Slides displaying optimal chromosome spreads were selected for oligo-FISH (Fluorescent *in situ* hybridization) using the oligo probes.

### Fluorescent *in situ* hybridization using oligo probes

The oligo-FISH protocol established by Braz et al., 2020 [36] was used with minor adjustments. Briefly, selected slides were pre-fixed with 4% formaldehyde for 15 minutes, followed by three washes with 2x SSC solution. Chromosomes on the slides were denatured at 85 ºC for 2 minutes. Subsequently, slides were incubated overnight with a hybridization mix containing 10 µL of 100% Formamide, 2 µL of Saline-Sodium Citrate (SSC) 20X, 4 µL of 50% Dextran, 1 µL of each oligo probe set (red and green) and 2 µL of dH^2^O at 37 ºC. Stringency washes with 2x SSC (Saline-sodium citrate) at 42 ºC for 10 minutes were performed post-hybridization. Images were captured using a ZEISS Axio Imager Z1 Apotome fluorescent microscope equipped with a Digital CCD AxioCam MRm camera and excitation/emission filters for DAPI (365/445 nm), Alexa488 (470/525 nm) and ROX (587/647 nm). At least five metaphases per species were analyzed to determine the distribution of probes on chromosomes. *Lens* species FISH images were processed using Adobe Photoshop 24.1.1. and were compared with the BLASTN pattern for both sets of oligo probes. Any deviations in the observed patterns relative to the BLASTN output were documented and correlated with the number of oligos in each probe at specific genomic regions, in an effort to explain the deviations.

## Results

### Oligo-FISH probe design strategy

**Fig 1:**
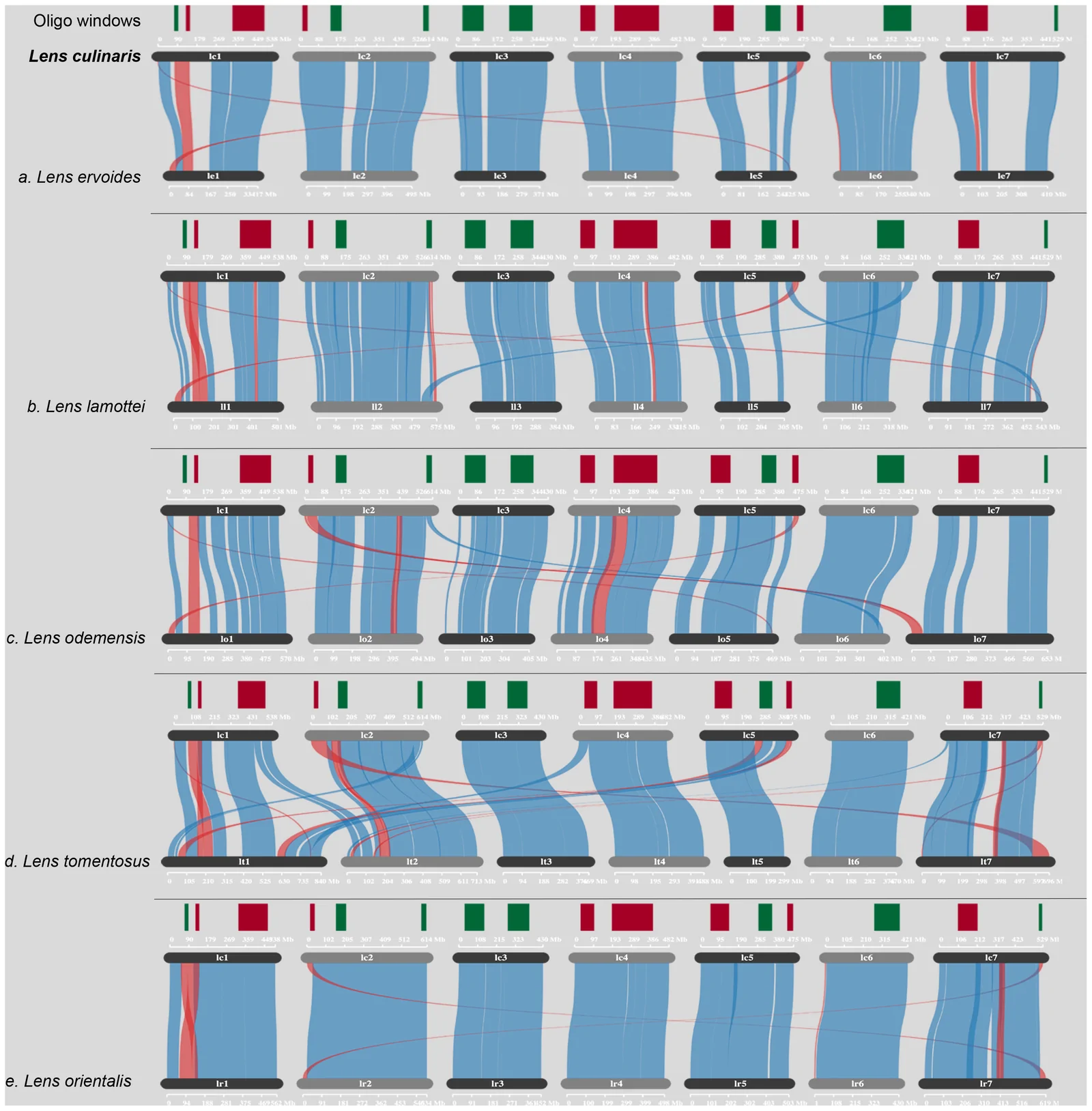
Synteny comparison between Lcu.2RBY chromosomes (Lc numbers; top in every pair) and those of each of five wild genomes. (a. Ler.1DRT, b. Lla.1ESP, c. Lod.1TUR, d. Lto.1BIG, e. Lor.1WPS). Green and red blocks on the top of each chromosome represent the predicted genomic windows in Lcu.2RBY, where probes would cover the maximum number of rearrangements relative to the wild genomes. Blue ribbons between chromosomes represent syntenic genomic regions, and light red ribbons represent inverted genomic regions.

The gene-based synteny presented in Fig 1 was the basis for the oligo probe design strategy. Synteny comparisons between the Lcu.2RBY genome assembly and the assemblies of the five wild genomes – Lor.1WPS, Lto.1BIG, Lod.1TUR, Lla.1ESP and Ler.1DRT, generated Lcu.2RBY rearrangement coordinates (S2 Table in S2 File) using the Synvisio (https://synvisio.github.io/) detailed visualization section. Lcu.1GRN and Lni.1VIC genomes were not assembled until after the initial oligo-probe design, so they were not included in the oligo-FISH probe design strategy. The rearrangements mapped to Lcu.2RBY leveraged coordinates that overlap the maximum number of rearrangements against the wild genomes (genomic windows), thus maximizing coverage of the Lcu.2RBY oligo probes (S3 Table in S2 File).

The final coordinates for each Lcu.2RBY genomic window are presented in Table 2 and Fig 1, colour-coded for each oligo pool (red and green). Each genomic window has at least 17 Mbp coverage, providing sufficient space for 3,375 oligos, each 45 nt in length. Additionally, each window is located at least 15 Mbp from chromosome ends, ensuring a safe distance from telomeric repeats, and is at least 28 Mbp away from adjacent windows; for these reasons, the chosen genomic windows (Table 2) differ from the overlapping rearrangement coordinates (S3 Table in S2 File). These measures ensure adequate resolution to differentiate between oligo probes, as proposed for Maize, which has larger chromosomes [25].

**Table 2:** Final selection of genomic windows for oligo probe development based on the Lcu.2RBY genome assembly. WS: Windows Start. WE: Windows End. Abbreviations reflect species names. Numbers reflect chromosome numbers/rearrangement numbers within the same chromosomes (Lor: *L. orientalis*; Lto: *L. tomentosus*; Lla: *L. lamottei*; Ler: *L. ervoides*; Lod: *L. odemensis*).

Lcu.2RBY chromosome	Lcu Start	Lcu End	Predicted Rearrangements captured	Windows Start	Windows End
1	78000000	90000000	Lor1_Inversion1	75500000	95000000
1	80000000	85000000	Lto1_Inversion1		
1	83500000	91600000	Lla1_Inversion1		
1	85000000	95000000	Ler1_Inversion1		
1	125000000	138000000	Ler1_Inversion2	131000000	150000000
1	126000000	140000000	Lor1_Inversion2		
1	131000000	138000000	Lla1_Inversion2		
1	440000000	445000000	Lto2_Translocation1	350000000	500000000
2	10000000	40000000	Lto7_Trans/Inv1	16000000	40000000
2	16500000	25000000	Lor7_Trans/Inv1		
2	114000000	120000000	Lto2_Inversion1	148000000	200000000
2	566000000	599000000	Lto1_Translocation1	584000000	610000000
2	584000000	605300000	Lod6_Translocation1		
3	–	–	No rearrangement	30000000	130000000
3	–	–	No rearrangement	250000000	360000000
4	2000000	50000000	Lto1_Translocation1	30000000	100000000
4	188000000	205000000	Lod4_Inversion1	190000000	400000000
4	235000000	250000000	Lod4_Inversion2		
5	–	–	No Rearrangement	50000000	145000000
5	280000000	291000000	Lto1_Trans/Inv1	295000000	365000000
5	330000000	366000000	Lto1_Translocation1		
5	442000000	447800000	Ler1_Trans/Inv1	442000000	472000000
5	444000000	451300000	Lla1_Trans/Inv1		
5	448000000	454300000	Lod1_Trans/Inv1		
6	–	–	No Rearrangement	250000000	380000000
7	–	–	No Rearrangement	95000000	195000000
7	509100000	515000000	Lto2_Trans/Inv1	508000000	525000000
7	509000000	516000000	Lor2_Trans/Inv1		

### Oligo-FISH probe predictions in *Lens* species

Oligo pools Lcu.2RBY_Gpool and Lcu.2RBY_Rpool are described in Table 3. Each contains 27,000 oligos, derived from 16 regions on the seven *L. culinaris* CDC Redberry chromosomes. Based on Lcu.2RBY predictions, the probes should result in 16 distinct FISH signals with 3,375 oligos each. Oligo probes were named based on their chromosomal location, e.g., gol11: green oligo chromosome 1, position 1. Gene annotation analysis showed that 16.98% of the oligos are within Lcu.2RBY genes, while the remaining are likely in intergenic regions.

**Table 3:** Lcu.2RBY oligo probes final coordinates and genomic information. Oligo probes are named according to their chromosomal position. gol11: green oligo chromosome 1 position 1.

Chromosome	Oligo probe	Start	End	Region Length	Number of oligos	Oligos/Kbp	Oligo pool
Lcu.2RBY.Chr1	gol11	76893900	78542425	1648525	3375	2.05	Lcu.2RBY_Gpool
Lcu.2RBY.Chr2	gol21	172423540	173547885	1124345	3375	3.00	
Lcu.2RBY.Chr2	gol22	604707040	605269325	562285	3375	6.00	
Lcu.2RBY.Chr3	gol31	32078260	33116187	1037927	3375	3.25	
Lcu.2RBY.Chr3	gol32	300905060	301516885	611825	3375	5.52	
Lcu.2RBY.Chr5	gol51	348012280	348539185	526905	3375	6.41	
Lcu.2RBY.Chr6	gol61	379187480	379713325	525845	3375	6.42	
Lcu.2RBY.Chr7	gol71	522206540	522577327	370787	3375	9.10	
Lcu.2RBY.Chr1	rol11	147086280	149067785	1981505	3375	1.7	Lcu.2RBY_Rpool
Lcu.2RBY.Chr1	rol12	355614280	356253425	639145	3375	5.28	
Lcu.2RBY.Chr2	rol21	36278340	36837580	559240	3375	6.03	
Lcu.2RBY.Chr4	rol41	6279460	6765987	486527	3375	6.94	
Lcu.2RBY.Chr4	rol42	304057720	304666605	608885	3375	5.54	
Lcu.2RBY.Chr5	rol51	57947160	58665547	718387	3375	4.7	
Lcu.2RBY.Chr5	rol52	468283260	468760065	476805	3375	7.08	
Lcu.2RBY.Chr7	rol71	160382620	161597887	1215267	3375	2.78	

**Fig 2:**
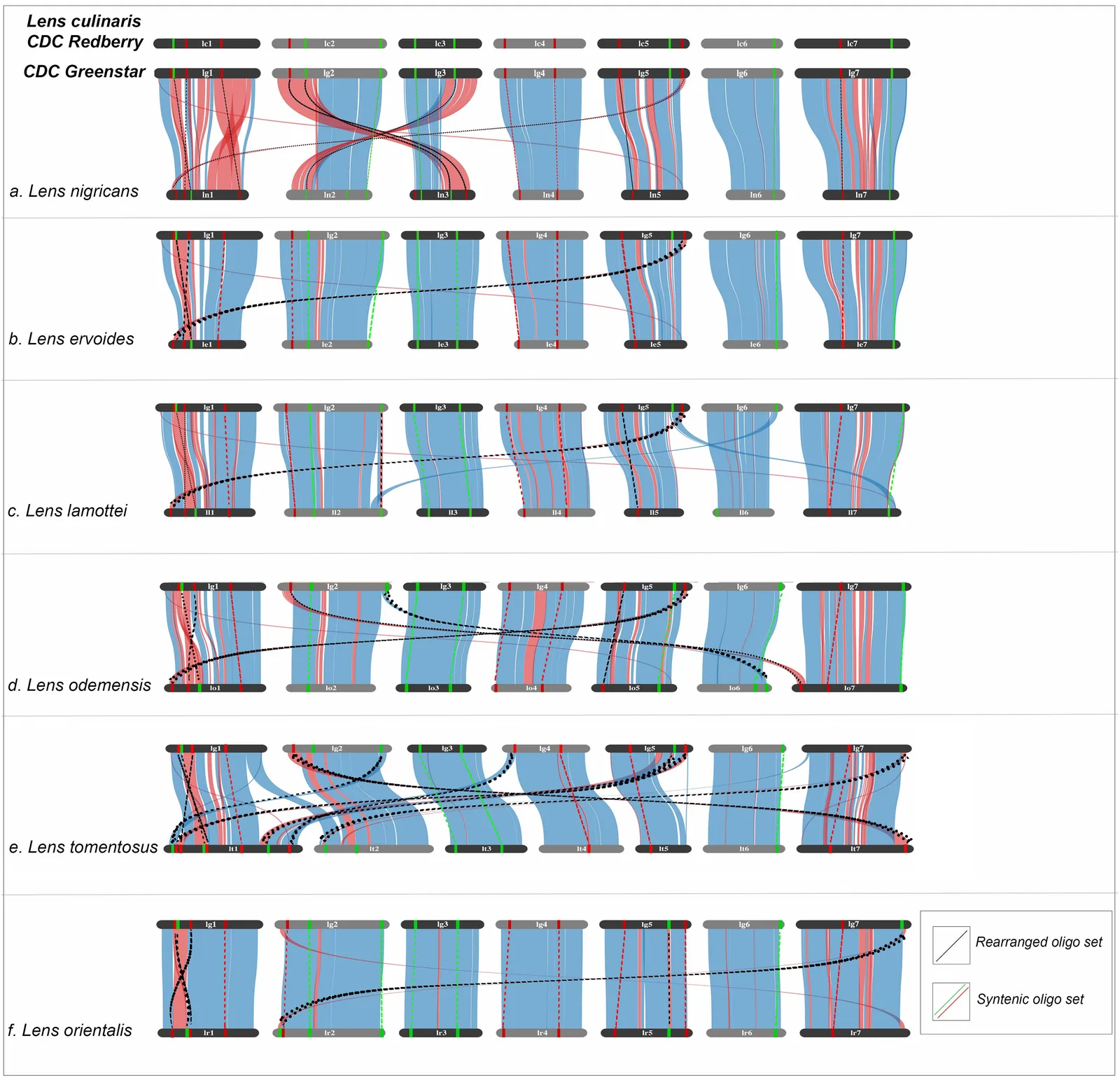
Oligo probes distribution according to BLASTN results in combinations of wild *Lens* genomes (Lni.1VIC, Ler.1DRT, Lla.1ESP, Lor.1WPS, Lod.1TUR, Lto.1BIG) relative to *Lens culinaris* CDC Greenstar (Lcu.1GRN). Lcu.1GRN is being used instead of Lcu.2RBY due to it having a higher assembly quality assessment. Black dashed lines highlight predicted rearrangements to be confirmed in oligo-FISH. Red and green dashed lines highlight syntenic oligo-FISH signals according to the predicted probe colour. Probe signals without dashed lines do not have homologous BLASTN hits.

As expected, based on the oligo probe design strategy, most Lcu.2RBY probes have a homologous BLASTN hit within each wild *Lens* genomes and Lcu.1GRN – every BLASTN hit was included as a predicted probe site. The average identity of both Lcu.2RBY oligo pools ranges from 96.21% for the *L. nigricans* genome to 99.79% for the *L. tomentosus* genome, with the highest number of mismatches for *L. nigricans* and the lowest for *L. orientalis* genomes (S4 Table in S2 File and Fig 2). The combination of the rearranged oligos (Fig 2 black dashed lines) and the syntenic oligos (Fig 2 coloured dashed lines) between the cultivated and the wild species provides a barcode for each chromosome of each of the analyzed accessions (S1 Fig in S1 File). Detailed probe visualization using JBrowse 2 [33] regarding probe origin in the Lcu.2RBY reference genome and homologs in wild species genomes can be accessed through https://knowpulse.usask.ca/experiment/Lens-oligo-FISH [37].

### Oligo-FISH can be used as a tool to evaluate genome assembly completeness: *Lens culinaris* CDC Redberry vs. CDC Greenstar

**Fig 3:**
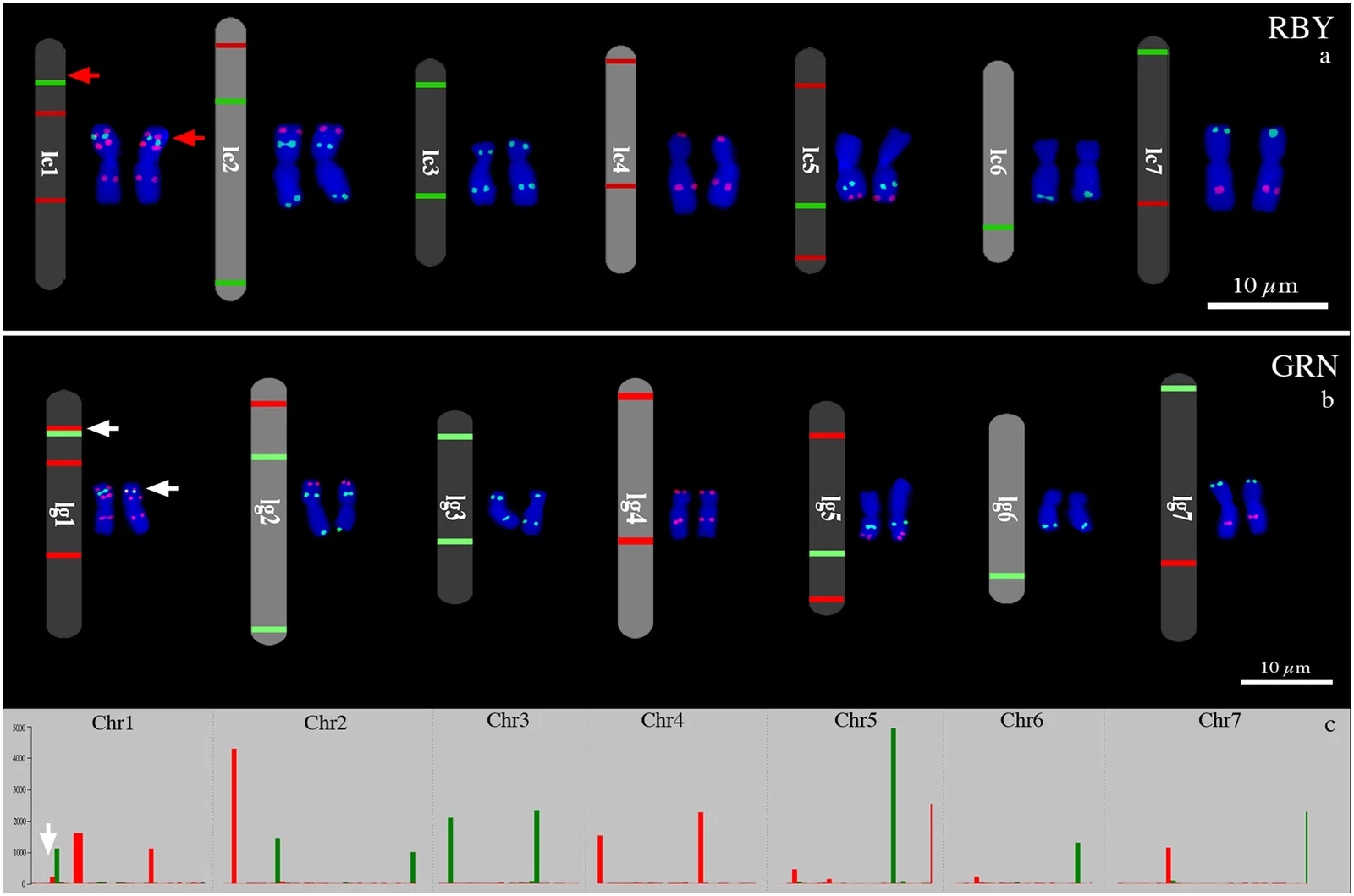
Oligo-FISH results for *Lens culinaris.* a. CDC Redberry (RB) and b. Greenstar (GS) compared to genome assembly oligo probe predictions (lc and lg predictions). c. Oligo probe density for CDC Greenstar assembly based on BLASTN results. Red arrows point to a missing and an extra red signal on Lcu.2RBY assembly and *in situ* chromosomes, respectively. White arrows point to homologous red signals both in Lcu.1GRN assembly and *in situ* chromosomes.

The predicted oligo distribution pattern for the Lcu.2RBY genome assembly (Fig 3a, lc#) matched the oligo-FISH results for chromosomes 2, 3, 4, 6, and 7. CDC Redberry chromosome 5 (*in situ*) is missing one red signal (rol51, red oligo chromosome 5, position 1) compared to Lcu.2RBY.Chr5 (lc5 assembly prediction), which is explained by a longer probe region and, consequently, a lower density of oligos for rol51 (Table 3). Rol51 does not have a high enough probe density to be visible in the FISH in comparison to other oligo probes on lc5.

*L. culinaris* CDC Redberry chromosome 1 showed an additional red signal *in situ* that was not predicted by the Lcu.2RBY.Chr1 assembly (lc1) (Fig 3a, red arrows). This signal was absent during oligo probe development. By contrast, *L. culinaris* CDC Greenstar—another genotype of the same species—displayed this additional red signal both *in situ* on chromosome 1 and in the Lcu.1GRN.Chr1 assembly (lg1) (Fig 3b and 3c, white arrows). The extra signal in CDC Greenstar corresponds to the first red oligo set from lc1 (rol11) (Table 3), which targets an additional homologous region, resulting in two distinct oligo-FISH signals (S2B Fig in S1 File, black arrow). Thus, the CDC Greenstar assembly more accurately represents *L. culinaris* chromosomes, providing a more complete genome assembly. For this reason, the Lcu.1GRN probe distribution was used as the reference for subsequent comparisons with wild *Lens* genomes (Fig 2).

### Genome assembly and synteny confirmations: oligo-FISH cross-species experiments

**Fig 4:**
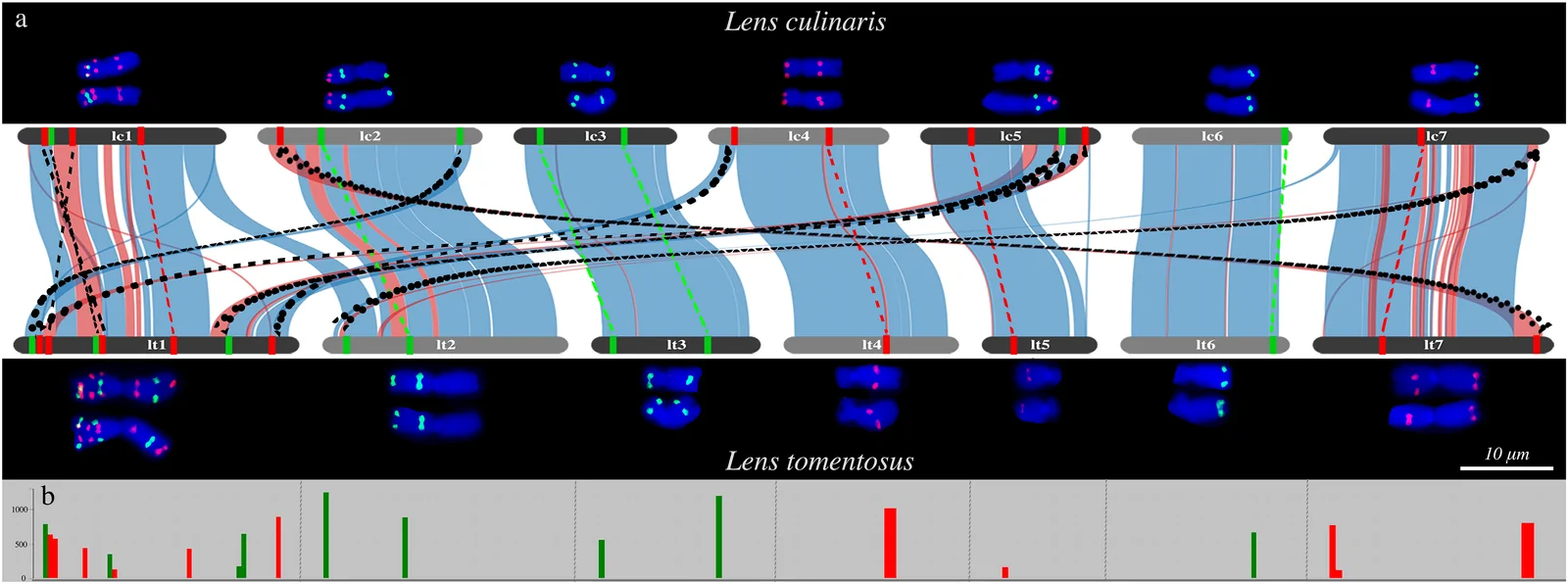
Oligo-FISH results compared to genome assembly oligo probe predictions for *Lens culinaris* CDC Greenstar (lg) and *Lens tomentosus* (lt). a. Oligo-FISH results alongside genome assembly oligo probe predictions. b. Oligo probe density in Lto genome assembly based on BLASTN analysis.

The Lcu.2RBY-based oligo probe sets used for FISH were successful in *L. tomentosus* (Fig 4) and the other five wild spp. (S3 Fig in S1 File). For most species, the predicted genome assembly oligo probe locations match the oligo-FISH signals, agreeing with the synteny comparisons in the wild genome assemblies. The *L. orientalis* (Lor) oligo-FISH signal pattern is similar to those of *L. culinaris* CDC Greenstar and CDC Redberry (Lcu) (S3A Fig in S1 File). BGE 016880 (Lor) chromosome 1 (lr1) possesses the extra homologous red signal present in both *in situ L. culinaris* accessions (rol11, see Table 3), which, in conjunction with the green signal gol11, shifts position relative to the stand-alone red probe rol12, confirming a major inversion on chromosome 1 of Lor relative to Lcu. The results also confirm a chromosome lr2-lg7 translocation in BGE 016880 (Lor) relative to Lcu.

All *L. tomentosus* (Lto) rearrangements matched the genome assembly predictions, confirming the proper chromosome assignment of scaffolds during the assembly process (Fig 4A and 4B). Lto has the most divergent probe pattern when compared to *L. culinaris,* with multiple translocations and inversions. These results confirm the high number of rearrangements predicted by the synteny analysis. Lto chromosome 1 has the extra rol11 signal seen in Lcu.1GRN and 2RBY oligo-FISH. The smaller size of Lto chromosome 5 allows the low-density red probe rol51 to be visible in the oligo-FISH (see Table 3) (Fig 4B), whereas it remains undetectable in Lcu or Lor.

Most *L. odemensis* (Lod) chromosomes match the predicted oligo probe pattern, confirming its genome assembly and synteny analysis. One exception is on chromosome 1, both *in situ* and lo1, which lack the extra split red signal seen in Lcu.1GRN (rol11). Chromosome 5 is also missing the low-density red signal (rol51), both *in situ* (S3B2 Fig in S1 File) and lo5. Chromosome 7 has one missing and one extra red signal that doesn’t match the BLASTN probe density (lo7). Based on our oligo-FISH analyses, we suspect potential assembly errors in this region (S3B Fig in S1 File).

*Lens lamottei* (Lla) has a similar probe distribution to Lod, with most chromosomes in the assembly confirmed through the oligo-FISH results (S3C Fig in S1 File). Chromosome 5 is missing rol51 *in situ* due to a low-density probe region (S3C2 Fig in S1 File). The same chromosome presents two extra green signals that are not present in the BLASTN probe density distribution (ll5), in which one of them is likely homologous to gol51 in Lcu.1GRN.Chr5 (lg5) (S3C2 Fig in S1 File). Chromosome 6 is missing a green signal, both *in situ* and ll6, which is present in the opposite chromosomal arm when compared to Lcu.1GRN (lg6).

*Lens ervoides* (Ler) has a similar probe pattern to Lla (S3D Fig in S1 File). Chromosome 5 is missing the red signal (rol51) present on the BLASTN (le5) due to a low-density region (see S3D2 Fig in S1 File). In the same chromosome, there is no oligo-FISH signal nor BLASTN hit homologous to the green gol51 that is found in Lcu.1GRN (lg5). The oligo sequence was likely lost during species differentiation since Ler is distantly related to Lcu in *Lens* spp. phylogeny [38,39].

*L. nigricans* (Lni) is the most distantly related species when compared to the reference Lcu [39] and is also the only species in which the oligo probes did not produce a clear pattern of signals (S4A Fig in S1 File) despite the assembly (Lni.1VIC) having been predicted to have high oligo density (S4B Fig in S1 File). When analyzing the distribution of these oligos for each Lcu.1VIC chromosome, there was a noticeably higher dispersion of probes, as compared to in the other *Lens* genomes, creating more non-specific signals, as seen in the oligo-FISH results. Oligos tend to be less conserved in distantly related species [25,40], reducing the likelihood of producing sufficient signals for microscope capturing and this is likely what is happening with this species.

### Precise *Lens* karyotyping and chromosome evolution

**Fig 5:**
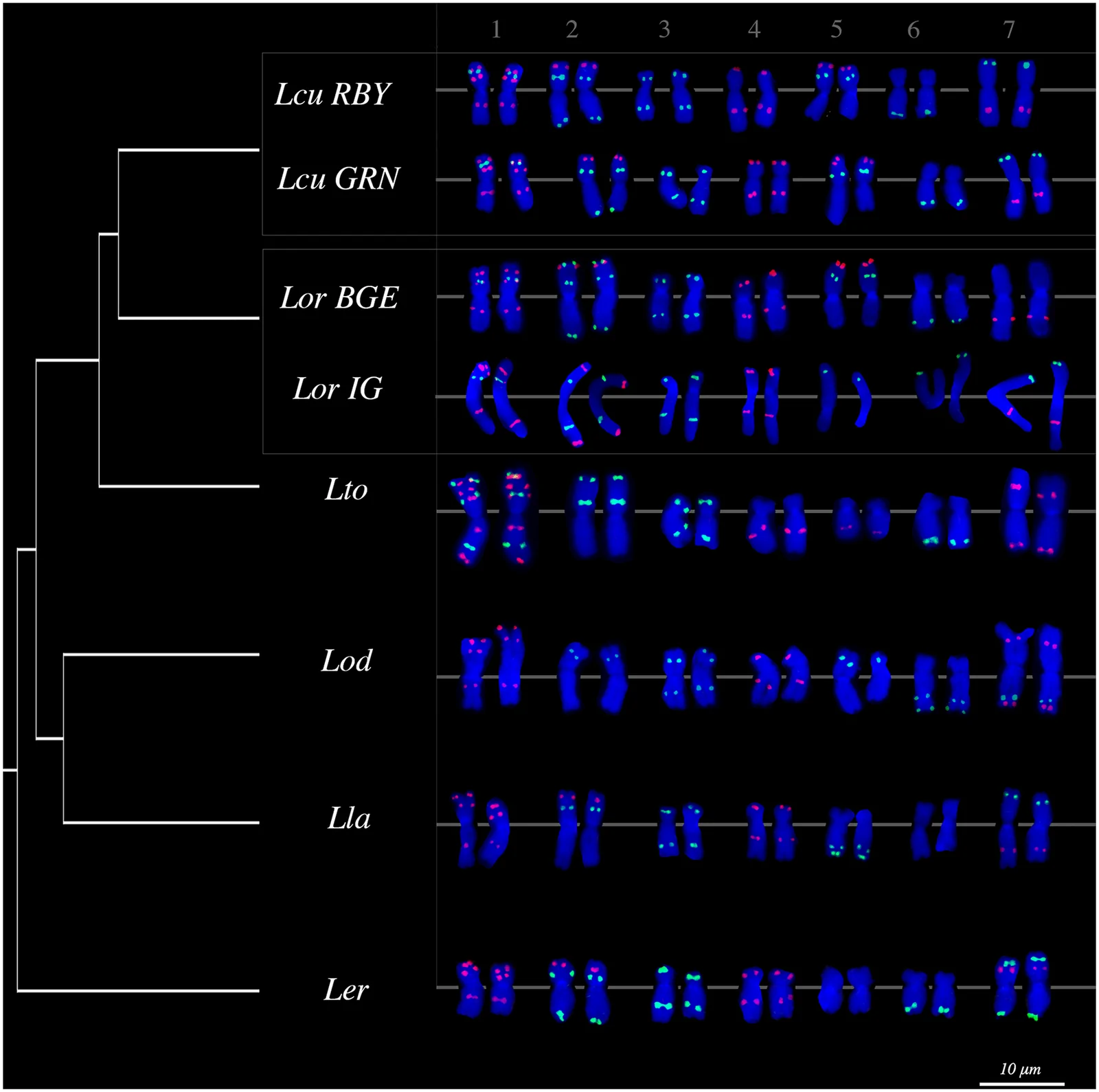
*Lens* species karyotype based on oligo-FISH signals. Species codes proposed as Table 1: Lcu: *L. culinaris*, Lor: *L. orientalis* (BGE016880 and IG72643), Lto: *L. tomentosus*, Lod: *L. odemensis*, Lla: *L. lamottei*, Ler: *L. ervoides*. Phylogeny proposed by Wong et al., 2015 [39].

The oligo probe patterns allow us to precisely define the homologous chromosomes in six of the seven analyzed *Lens* spp., building a genus-wide karyotype system (Fig 5). *L. culinaris* and *L. orientalis* possess a similar karyotype. Both species have 16 oligo-FISH signals on their chromosomes (Table 4), highlighting their sequence homology. The arrangement of those probes reflects the structural differences they do have, notably on chromosomes 2 and 7. *L. tomentosus* also has a similar number of oligo-FISH signals compared to Lcu and Lor, with a single addition of the rol51 probe, which was not dense enough to visualize in the other species. These results attest to the higher level of sequence homology among closely related *Lens* spp. (Table 4).

**Table 4:** Number of oligo-FISH signals in each *Lens* species’ chromosomes. Species are organized according to gene pool classification as proposed by Wong et al., 2015 [39]. n total: total number of oligo-FISH signals.

Species	Gene Pool	Chr1	Chr2	Chr3	Chr4	Chr5	Chr6	Chr7	*n total*
*L. culinaris*	1	4	3	2	2	2	1	2	*16*
*L. orientalis*	1	4	4	2	2	2	1	1	*16*
*L. tomentosus*	1	8	2	2	1	1	1	2	*17*
*L. odemensis*	2	3	1	2	2	1	2	3	*14*
*L. lamottei*	2	3	2	2	2	2	0	2	*13*
*L. ervoides*	3	3	3	2	2	0	1	3	*14*

Despite the close phylogenetic relatedness of *L. tomentosus* to both Lcu and Lor, this is not obvious when using Lto oligo-FISH signal arrangements, as they differ drastically from the other two species in chromosomes 1, 2, 4, 5 and 7. Lto chromosome 1, the most rearranged chromosome relative to all other *Lens* spp., has double the number of oligo-FISH signals when compared to Lcu and Lor chromosomes 1 (Fig 5, Table 4). This concentration of probes in one chromosome highlights how the remaining chromosomes of Lto are affected due to the genomic changes. For example, chromosome 5 is visibly smaller compared to that of other species.

*L. odemensis* has a similar karyotype in probe signal, number and arrangement to *L. lamottei* (Fig 5, Table 4). The similar probe signal number reflects a higher sequence homology among these two species rather than with species from the primary gene pool (Lcu, Lor and Lto). Lod and Lla differ in chromosomes 2, 5, 6 and 7 by five oligo signals (Fig 5, Table 4). *L. ervoides* also has a similar karyotype to Lod and Lla, with 14 oligo-FISH signals, although the species is evolutionarily distant [39]. Chromosomes 1, 3 and 4 are identical in oligo-FISH signals among Ler, Lod and Lla. The reduction of oligo-FISH signals according to *Lens* species differentiation (Table 4) is related to the oligo sequence divergence from *L. culinaris* – the oligo reference – to the more diverged species such as Lod, Lla and Ler [36,40,41].

### Oligo-FISH probes can be used to detect intraspecies structural variation in *Lens* species

**Fig 6:**
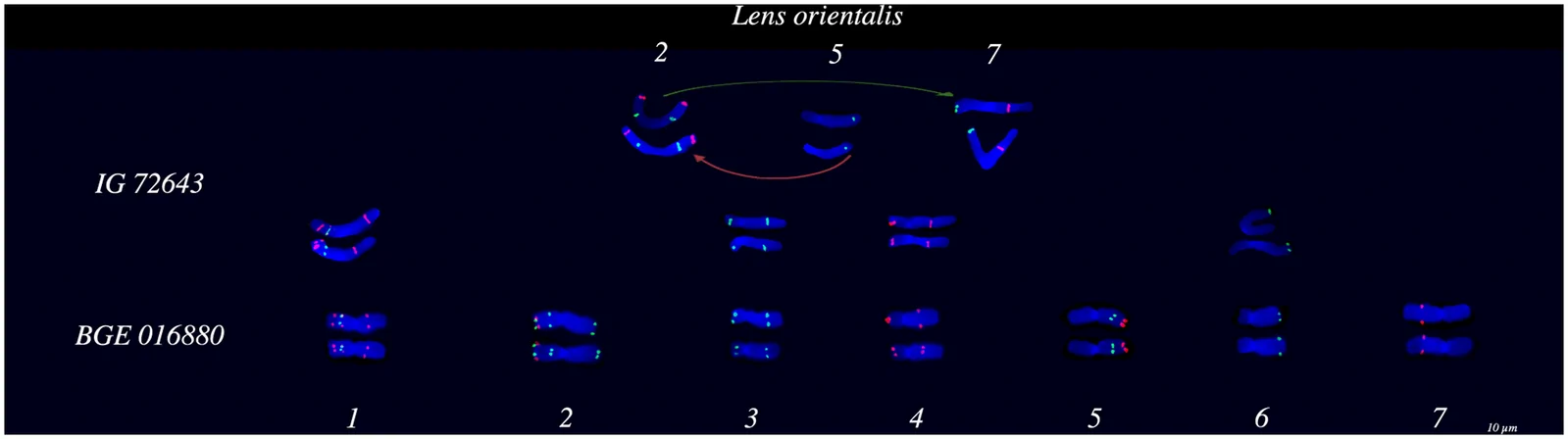
Oligo-FISH analysis of *Lens orientalis* IG 72643 in comparison to BGE 016880. The green arrow points to a proposed translocation between chromosomes 2 and 7. The red arrow points to a proposed translocation between chromosomes 5 and 2.

*L. orientalis*, the closest relative to *L. culinaris,* is often used in plant breeding programs for the introgression of beneficial alleles from the wild parent [9]. The Lcu.2RBY oligo probes were applied to a second accession of *L. orientalis,* IG 72643, and compared with BGE 016880 – the first *L. orientalis* accession used in this study and the only one with an assembled genome (Fig 6).

Chromosomes 1, 3, 4 and 6 of IG 72643 have identical probe distribution when compared to BGE 016880, while chromosomes 2, 5 and 7 differ between the two accessions. Chromosome 2 has one extra red signal and a missing green signal in IG 72643, chromosome 5 is missing one red signal, and chromosome 7 has an extra red signal. The arrangement of those probes is different only in IG 72643 chromosome 2.

It is possible to propose rearrangements that led to the karyotype differentiation between these two accessions. A translocation between chromosomes 2 and 7 could lead to the extra green signal in chromosome 7 (Fig 6, green arrow), while another translocation between chromosomes 5 and 2 could lead to the extra red signal in chromosome 2 (Fig 6, red arrow). Those rearrangements will be further confirmed after finalizing the genome assembly of IG 72643 and performing synteny analysis with BGE 016680.

## Discussion

Oligo-FISH studies across multiple species primarily rely on the genome assembly of a single species, with the probes then utilized in related species lacking a genome assembly to detect structural variation [25,27,40,42]. We developed an oligo-FISH probe system for karyotyping across the genus *Lens,* starting with a cultivated *L. culinaris* genome assembly (Lcu.2RBY) (Table 3), followed by synteny analysis and homology filtering against the genome assemblies of five *Lens* spp*.* wild relatives (Figs 1 and 2).

Advancements in genome sequencing technologies are supporting the cost-effective development of high-quality genome assemblies; however, limitations still exist, primarily related to the assembly of large, highly repetitive genomes, such as those of *Lens* spp. [15]. Ultra-long-read sequencing technologies and contact mapping have helped but still require a large investment in sequencing to achieve the required depth to be confident in an assembly. The accuracy of synteny analysis can be affected when comparing relatively large genomes like those of *Lens* spp., among themselves. The use of complementary strategies, such as cytogenetic maps, for validating the genome assembly and synteny analysis, is thus highly recommended [43,44].

Our *Lens* oligo-FISH system innovatively enables the validation of proper genome assembly scaffolding into chromosomes and confirms the proposed synteny among *Lens* spp. Oligo-probe-based genomic predictions match most of the *Lens* physical chromosomes, attesting to the quality of the genome assembly scaffolding process, even in highly repetitive genomes [15]. The changes in most probe positions for *L. culinaris* and its wild relatives confirm the rearrangements detected in the synteny analysis (Fig 5 and S3 Fig in S1 File). The probes are also useful for suggesting improvements to the assembly of specific chromosomes, such as Lod.1TUR chromosome 7, where the predictions do not match the oligo-FISH signals due to misassembled regions (S4B Fig in S1 File). Differences in the predictions of oligo probes for Lcu.1GRN and Lcu.2RBY, which were then confirmed with the oligo-FISH, were used for quality assessment, attesting to a likely unassembled region on CDC Redberry chromosome 1 (Fig 3). Lou et al. (2014) reported the use of oligo-FISH painting probes to assess genome assembly completeness in *Cucumis* species using meiotic pachytene cells.

The oligo probes enable us to propose a precise karyotype for six *Lens* spp., accurately defining each pair of chromosomes without the need for chromosome measurements or the use of repetitive DNA FISH probes. Multiple rearrangements have already been proposed among *Lens* spp. [4,21–23,45–48]. These have now been physically and precisely confirmed in our work. These rearrangements which we have characterized through oligo-FISH hybridization patterns show how species of *Lens* have diverged while retaining substantial sequence similarity. We also confirm the limitation of oligo-FISH probes when conducting experiments with more distantly related species [36,40,49], with the lack of clear signals on *L. nigricans* chromosomes. We have detected intraspecific variation in two different genotypes of *L. orientalis,* documenting how crossing barriers to cultivated *L. culinaris* may vary even in its immediate wild relative. The occurrence of distinct structural rearrangements and different karyotypes within these species had already been proposed [16–18,45,47].

The oligo-FISH probes are useful for characterizing and detecting structural variation among genotypes of the same or different *Lens* spp. in the absence of a genome assembly. This allows researchers to screen wild relatives not just for phenotypes of interest, but also to select accessions that are more likely to lead to successful pairing and recombination during F1 meiosis. Crossings between species from different gene pools can bring undesirable alleles into the cultivated *L. culinaris* genome due to linkage drag associated with the numerous rearrangements [15]. The probes would be useful for screening populations and tracking specific chromosome combinations, allowing breeders to stack desirable alleles while reducing linkage drag issues. Chromosome 7 is an example of the linkage challenges caused by rearrangements when trying to transfer alleles due to structural changes; Cao et al. (2024) demonstrated extensive linkage drag on this chromosome in interspecific RILs populations derived from *L. culinaris* crosses with one *L. orientalis* accession.

The developed oligo-FISH probe system in *Lens* spp. is useful for assessing genome assembly completeness pertaining to chromosome structure and composition, as well as for further screening of populations and intra/interspecific hybrids in plant breeding programs. Meiotic chromosome behaviour during reproduction when facing larger structural variation is also of interest for crop production and genome evolution studies. Our cross-species oligo-FISH system enables future studies in the *Lens* genus regarding chromosome pairing, recombination and hybrid viability for six of the seven species. This is a significant achievement for legume science and breeding, with an oligo-FISH system that sheds light on the larger structural variation of legume’s genomes.

## Supporting information

S1 File Supplementary figures. File containing supplementary figures.
(DOCX)

S2 File Supplementary tables. File containing supplementary tables.
(XLSX)
