# Supplementary material for: Comparative oligo-FISH validates genome assemblies and delivers a precise karyotype for Lens Mill. species
Source: PLoS One. 2026 Sep 21;21(9):e0354141. doi: 10.1371/journal.pone.0354141 (PMC13592646; doi:10.1371/journal.pone.0354141)
Supplement: Supplementary file 1 [file pone.0354141.s001.docx]

**S1 Fig. Oligo barcode system based on BLASTN results for each *Lens* species chromosome.** The middle section of each chromosome is referenced as “:”.

**S2 Fig. Lcu.2RBY (A) and Lcu.1GRN (B) red probe distribution.** The x-axis represents *in silico* chromosomes, and the y-axis represents red oligo probe enrichment per 1 Mbp. The black arrow highlights the origin of the extra red signal on Lcu.1GRN.Chr1 as Lcu.2RBY.Chr1.

**S3 Fig. Oligo probe prediction, density and oligo-FISH results in Lens culinaris compared with wild Lens species.** .1 Lens culinaris CDC Greenstar oligo-FISH and probe prediction .2 Wild genomes oligo probe density based on BlastN results.

**S4 Fig. Lens nigricans Oligo-FISH highlight the lack of a clear pattern of signals for both Lcu.2RBY oligo probes.** A: Oligo-FISH images. B: BlastN probe density for Lni.1VIC chromosomes.
